# The Prognostic Values of PARP-1 Expression in Uveal Melanoma

**DOI:** 10.3390/cells10020285

**Published:** 2021-01-31

**Authors:** Malgorzata Gajdzis, Stamatios Theocharis, Jerzy Klijanienko, Nathalie Cassoux, Sophie Gardrat, Piotr Donizy, Radoslaw Kaczmarek, Pawel Gajdzis

**Affiliations:** 1 Department of Ophthalmology, Wroclaw Medical University, 50-556 Wroclaw, Poland; radoslaw.kaczmarek@umed.wroc.pl; 2 First Department of Pathology, National and Kapodistrian University of Athens, 15772 Athens, Greece; theocharis@ath.forthnet.gr; 3 Department of Pathology, Curie Institute, 75005 Paris, France; jerzy.klijanienko@curie.fr (J.K.); pawel.gajdzis@umed.wroc.pl (P.G.); 4 Department of Ophthalmology, Institut Curie, 75005 Paris, France; nathalie.cassoux@curie.net; 5 Department of Biopathology, Institut Curie, PSL Research University, 75005 Paris, France; sophie.gardrat@curie.net; 6 Department of Pathomorphology and Oncological Cytology, Wroclaw Medical University, 50-556 Wroclaw, Poland; piotrdonizy@wp.pl

**Keywords:** PARP, PARP-1, uveal melanoma, immunohistochemistry

## Abstract

Background: Uveal melanoma is the most common primary intraocular malignancy in adults. In advanced cases, the prognosis is very poor. Thus far, no effective methods of pharmacotherapy of this cancer have been found. The aim of the study was to evaluate the expression of PARP-1, the best-known member of the family of poly(ADP-ribose) polymerases, in uveal melanoma and its associations with clinicopathological parameters, overall survival, and disease-free survival. Methods: The study included 91 patients who underwent enucleation due to uveal melanoma. PARP-1 expression was assessed by immunohistochemistry. Results: High PARP-1 expression was associated with more frequent chromosome 3 loss, higher histopathological grade, bigger tumor size, and absence of intrascleral extension. High PARP-1 expression was associated with shorter overall survival time and disease-free survival time. Conclusions: The above findings indicate that high expression of PARP-1 can be considered as an unfavorable prognostic factor in uveal melanoma.

## 1. Introduction

Uveal melanoma (UM) is the most common primary intraocular malignancy in adults. With a mean age-adjusted incidence of 5.1 cases per million per year, it appears to be a rare disease, but is important in ophthalmic practice [1]. The reason is the high tendency to metastasize—about 50% of patients develop metastasis, often many years after the treatment of the primary tumor [2]. Five-year survival rates vary considerably depending on the stage of the disease [3]. However, in the case of systemic disease, the prognosis worsens dramatically. Only 8% patients with metastatic melanoma survive two years [4]. The meta-analysis of 29 studies conducted during 1988–2015 proved how serious the problem is in the treatment of UM. It showed that the mean progression-free survival and overall survival did not change over the years, regardless of the treatment method used [4]. For this reason, numerous studies are being conducted looking for new prognostic factors as well as potential new therapeutic options.

Poly(ADP-ribose) polymerases (PARP) are a family of proteins involved in a number of cellular processes. These enzymes are present in all eukaryotes except yeast [5]. Of the 17 known PARP homologs in human, PARP-1 is the best known and most extensively studied. The most important function of PARP-1 is participation in the DNA repair processes [5]. In the case of minor DNA damage, the enzyme detects damaged areas and participates in the chromatin remodeling process [6,7]. Moreover, PARP-1 recruits other proteins to DNA damage sites [8,9,10]. Thus, it engages in such DNA repair mechanisms as base excision repair (BER), nucleotide excision repair (NER), DNA mismatch repair (MMR), and maintenance of replication fork stability [11]. It also participates in non-homologous end-joining and homologous recombination—pathways that ensure the repair of DNA double-strand breaks [12,13]. At moderate levels of DNA damage, PARP-1 activation results in cleavage of PARP-1 by caspases into two fragments, which is a hallmark of apoptosis and might modulate the apoptotic process [14]. In the case of severe DNA damage, over-activation of PARP-1 occurs, leading to programmed necrotic cell death [15].

It is also worth emphasizing that PARP-1 is one of the main nicotinamide adenine dinucleotide (NAD) consuming enzymes. NAD is an essential cofactor of redox enzymes and a substrate for enzymes that regulate DNA repair, gene expression, and the stress response [16]. Changes in PARP-1 expression, both during the course of the disease and during treatment with PARP inhibitors, may thus have a much more complex effect on cell metabolism.

Apart from the important role in DNA repair, PARP-1 also participates in the regulation of inflammatory processes. PARP-1 promotes inflammatory responses by positively regulating the pro-inflammatory Nuclear Factor-κB (NF-κB) transcription factors [17]. NF-κB activation pathways are involved not only in inflammation but also in immunity and carcinogenesis and can lead to tumor cell proliferation, invasion, angiogenesis, and metastasis [18].

The enzyme—involved in numerous DNA repair processes, regulating cell death in the event of excessive damage, and additionally affecting chronic inflammation—must be of interest in the context of carcinogenesis. For this reason, in recent years, we observed an increase in the interest of PARP-1. There are numerous studies on the mechanisms of action and the possibility of using its expression in the assessment of prognosis in various diseases, particularly in cancer. It is also being investigated as a target point for therapy. In this study, we examined the expression level of PARP-1 in primary UM. The aim of the study was to assess whether the degree of expression could be a prognostic factor in this relatively rare but deadly cancer. Additionally, we assessed if PARP-1 could be useful for molecular-targeted therapy.

## 2. Materials and Methods

### 2.1. Patients

Medical records and archive histopathological specimens of 91 patients with primary UM diagnosed during 2007–2008 at the Curie Institute, Paris, France were used in the study. All patients underwent enucleation as primary treatment. Patients with prior radiotherapy or chemotherapy were excluded from the study. Cases with too small amount of tumor cells in paraffin-embedded tissue were also excluded. The documented follow-up period was up to 115 months.

In our study, we considered clinicopathological parameters that are well-known prognostic factors in UM: chromosome 3 loss, presence of metastasis, mitotic activity, grading, intra- or extra-scleral extension, ciliary body involvement, tumor size, and patients’ age. Chromosome 3 loss analysis was assessed by CGH (comparative genomic hybridization), FISH (fluorescence in situ hybridization), or karyotype studies. It was available for 67 patients. Mitotic activity was assessed on X400 in 40 fields using hematoxylin and eosin staining. It was determined in 89 cases—in the remaining two cases, there was too much melanin in the tumor cells, preventing reliable evaluation. The histological grading was based on conventional criteria: G1, spindle cell uveal melanoma (>90% spindle cells); G2, mixed cell uveal melanoma (>10% epithelioid cells and <90% spindle cells); and G3, epithelioid cell uveal melanoma (>90% epithelioid cells). Other data—presence of metastasis, tumor size, age, and tumor localization—were obtained from the medical documentation.

The study was conducted in accordance with the Declaration of Helsinki, and it was approved by the Institutional Review Board of the Wroclaw Medical University, Poland (KB-508/2019—approval for statutory subsidy). Due to the retrospective nature of the studies and the lack of impact on the treatment of patients, it was not necessary to obtain informed consent.

### 2.2. Immunohistochemistry

Immunohistochemical studies were performed on the tissue materials fixed in 10% buffered formalin and embedded in paraffin blocks. Freshly prepared tissue sections (4 µm thick) were deparaffinized, rehydrated and then subjected to epitope retrieval. Commercially available antibodies against PARP-1 (clone: sc-74470 (B10), dilution: 1:50, Santa Cruz Biotechnology, Santa Cruz, CA, USA) and Autostainer 48 (DAKO) were utilized for immunohistochemical staining. Liquid Permanent Red (DAKO) was used as detection system. Red chromogen enabled visualization in tissues containing a large amount of melanin. As a positive control, human placenta and cutaneous melanoma tissues were used, as recommended by the manufacturer. The negative (isotype) controls were processed using FLEX Mouse Negative Control (DAKO).

**Figure 1:**
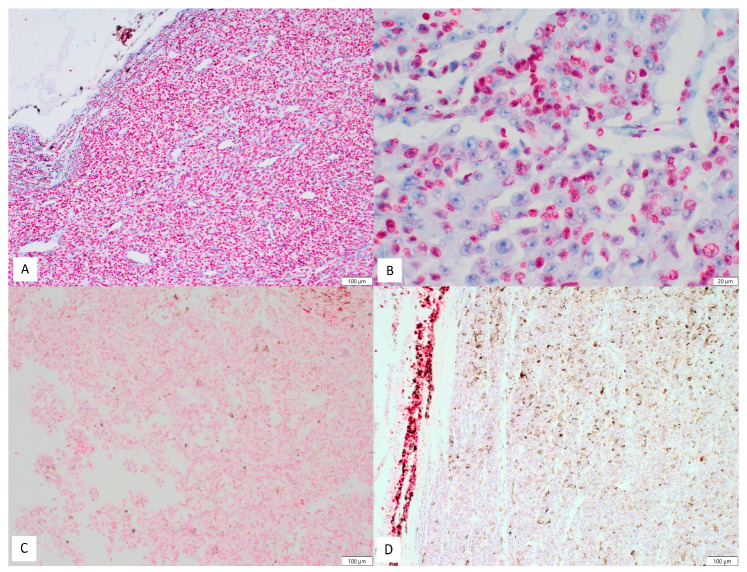
Examples of PARP-1 staining in uveal melanoma cells: (**A**) example of high expression (100×); (**B**) example of high expression—intense nuclear staining visible only in about half of tumoral cells (400×); (**C**) example of low expression (100×); and (**D**) complete lack of staining in tumoral cells. Focally positive internal control (100×).

Expression of PARP-1 was assessed by two independent pathologists (P.G and P.D.) with full agreement, based on observation of at least 1000 cells in each case. Only nuclear expression was considered positive. In some cases, there were also weak cytoplasmic expression in the tumoral cells, but it was interpreted as nonspecific and nondiagnostic. Scoring of PARP-1 immunostains was done using a semi-quantitative scale of the ImmunoReactive Score (IRS) [19,20]. It is expressed as multiplication between positive cells proportion score (0–4) and staining intensity score (0–3). For percentage of positive cells: 0 = no positive cells; 1 = <10% positive cells; 2 = 10–50% positive cells; 3 = 51–80% positive cells; and 4 = >80% positive cells. For intensity of staining: 0=no color reaction; 1 = mild reaction; 2 = moderate reaction; and 3 = intense reaction. The final, integrated scores ranged 0-12 [21]. For the purposes of statistical analysis, the cut-off point was set at ≥4 (low expression for 0-3 and high expression for 4–12). Examples of PARP-1 staining in tumoral cells are presented in Figure 1A–D. The determination of the cut-off point resulted from the statistical analysis as well as literature review on PARP-1 expression. The cut-off point for percentage of positive cells was set at ≥3 (low percentage for 0–2 and high percentage for 3–4). For intensity of staining, the cut-off point was set at ≥2 (low intensity for 0–1 and high intensity for 2–3).

### 2.3. Statistical Analysis

To assess the associations between PARP-1 expression and clinicopathological factors, Chi-square and U Mann–Whitney test were used. Overall survival curves and disease-free survival curves were constructed using the Kaplan–Meier estimator. The differences between the curves were compared on the basis of the log-rank test. A p-value less than or equal to 0.05 was considered statistically significant. All statistical analyses were performed in the Statistica 13 software (StatSoft Polska, Krakow, Poland).

## 3. Results

Tissue materials from tumors from 91 patients (including 55 women and 36 men) were used in the study. The average age was 63 years. Clinicopathological characteristics of the study group are presented in Table 1. Low PARP-1 expression was noted in 35 cases (38.5%), while high in 56 cases (61.5%). The intensity of the reaction was described as low in 32 cases (35%) and high in 59 (65%). The percentage of positive cells was low in 47 cases (52%) and high in 44 (48%).

The high intensity of the reaction was statistically significantly associated with the male gender (*p* = 0.036), absence of intrascleral extension (*p* = 0.025), bigger tumor size (*p* = 0.039), and more frequent chromosome 3 loss (*p* = 0.017). Table 3 summarizes the results for statistically significant correlations of reaction intensity.

A high percentage of positive cells were associated with bigger tumor size (*p* = 0.036), higher histopathological grade (*p* = 0.024), and more frequent chromosome 3 loss (p=0.033). Table 4 summarizes the results for statistically significant correlations of percentage of positive cells. 

**Figure 2:**
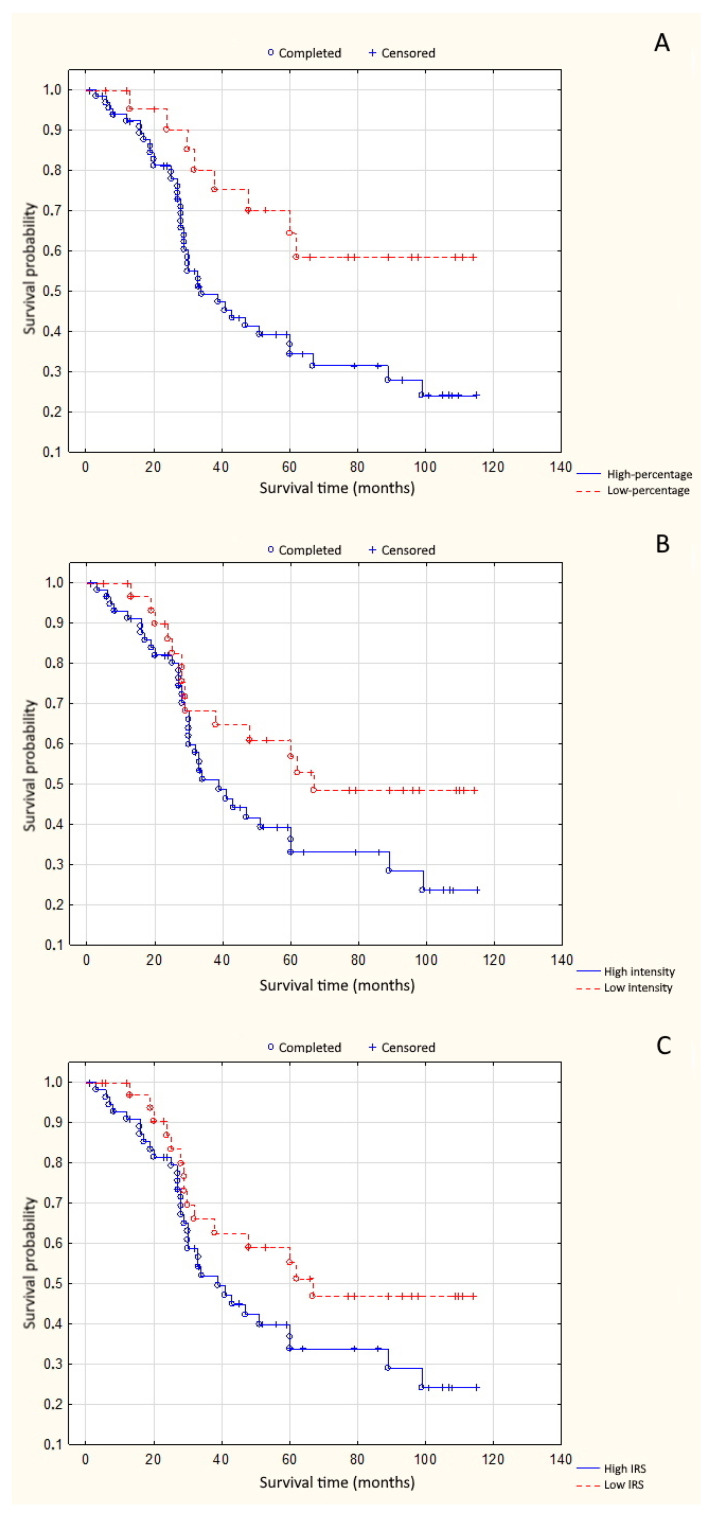
(**A**) Correlation between PARP-1 percentage of positive cells and overall survival time (*p* = 0.008); (**B**) correlation between PARP-1 reaction intensity and overall survival time (*p* = 0.072); and (**C**) correlation between PARP-1 total expression and overall survival time (*p* = 0.089).

**Figure 3:**
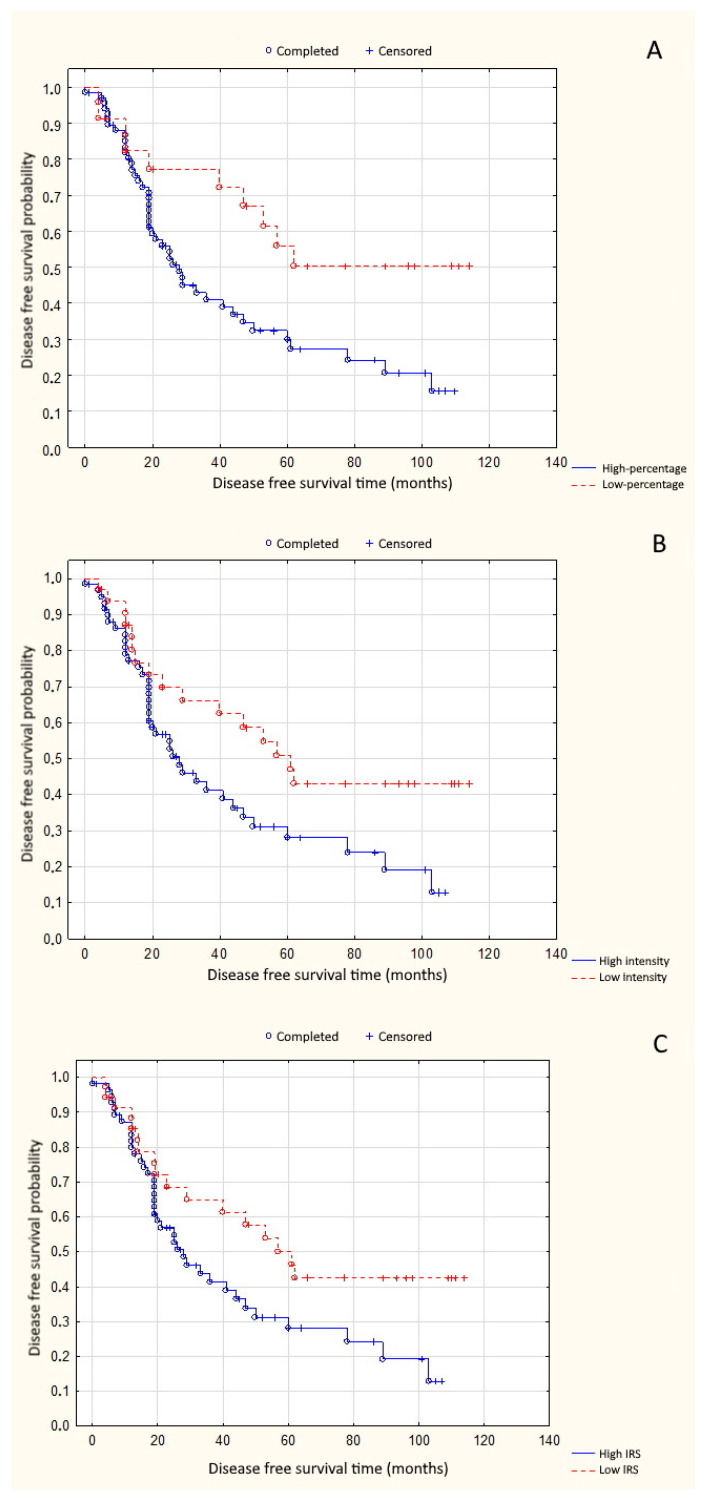
(**A**) Correlation between PARP-1 percentage of positive cells and disease-free survival time (*p* = 0.012); (**B**) correlation between PARP-1 reaction intensity and disease-free survival time (*p* = 0.028); and (**C**) correlation between PARP-1 total expression and disease-free survival time (*p* = 0.039).

High percentage of positive cells was significantly associated with shorter overall survival time (*p* = 0.008) (Figure 2A). High reaction intensity tended to decrease the likelihood of overall survival (OS) in patients with uveal melanoma (*p* = 0.072) (Figure 2B). A similar association occurred in the case of total high expression of PARP-1 (IRS ≥ 4) (*p* = 0.089) (Figure 2C). High percentage of positive cells, high reaction intensity, and total high expression significantly shortened disease-free survival time (DFS) (*p* = 0.012, *p* = 0.028, and *p* = 0.039, respectively) (Figure 3A–C).

## 4. Discussion

Uveal melanoma is a relatively rare disease, but it is a significant problem in ophthalmic practice, especially due to the high mortality rate and the lack of effective treatment methods in the advanced stage. New prognostic factors are constantly being sought to more precisely define the risk of disease progression. It is also important to look for new target points for therapy. An additional problem is that the biology of uveal melanoma significantly differs from that of cutaneous melanoma. For this reason, it is not possible to use regimens that work well in cutaneous melanoma in uveal melanoma. In recent years, PARP-1 functioning in different diseases became an attractive research area. The role of PARP-1 in the processes of DNA repair and regulation of inflammation makes the determination of its expression particularly useful in cancer and autoimmune diseases.

Several studies have shown that many tumors exhibit an elevated level of PARP-1 expression, and overexpression is often associated with disease progression. Moreover, loss of PARP-1 in mouse models may decrease tumor development [17]. Possibly PARP-1 inhibition can suppress damaged DNA repair and improve tumor killing. On the other hand, PARP-1 knockout mice after exposure to radiation damage showed an increased risk of developing other tumors—in the cerebellum and the skin. This is because PARP-1 inhibition may unmask recessive mutations in tumor suppressor genes [22]. Moreover, PARP-1 may have an opposite effect on the development of the disease, depending on the mechanism of its formation. For example, PARP-1 can protect against colorectal tumor formation and at the same time promoting inflammation-driven progression of these tumors [23]. Another aspect is the importance of PARP-1 expression depending on the severity of the disease. One study in gastric cancer patients showed that high PARP-1 expression was associated with poor prognosis, but only in patients with a more advanced disease stage. Patients with an advanced TNM stage (III–IV) and high PARP-1 expression had significantly reduced DFS and OS. This association was not present in patients with early TNM stage (I–II) [24]. Another relationship was found in breast cancer. High PARP-1 expression was correlated with poor prognosis in lymph node negative early breast cancer [25,26]. In addition, in more advanced stages of breast cancer, high PARP-1 expression helped in predicting survival: it correlated with shorter DFS and OS and increase risk of recurrence [27].

The tissues used in our study were obtained from eyes after enucleation. This procedure is performed only in advanced disease cases [4]. Therefore, in the studied cases, we showed a significant relationship between OS and DFS in patients with advanced UM. Investigating whether PARP-1 expression in cases of early UM affects the prognosis is problematic, because, due to the small size of the tumor and its frequent location in the posterior part of the eyeball, obtaining tissues for histopathological examination is practically impossible.

There are many reports on the possible use of PARP-1 expression as a prognostic factor in various neoplasms, in which the severity of the disease does not play a significant role. Thus, an increased level of PARP-1 expression was observed in lung, prostate, uterus, and ovarian cancers [28,29]. High PARP-1 expression alone and in combination with the expression of other DNA repair molecules (γH2AX, BRCA1, and BRCA2) is prognostic factor for shorter survival in soft tissue sarcoma patients [30]. PARP-1 expression is also increased in colon adenoma and carcinoma [31]. The evaluation of PARP-1 expression as a prognostic factor in mucosal melanomas has also been shown [32]. Our results are in line with the general trend. The demonstrated associations with recognized prognostic factors—loss of chromosome 3, tumor size, and histopathological grade—indicates that high PARP-1 expression can be considered an unfavorable prognostic factor in UM.

There are only a few reports in the literature published so far on the role of PARP-1 in uveal melanoma. In one study, a small group of tumors (n = 12) were examined and PARP expression was observed with different intensity [33]. Another study analyzed PARP-1 expression in the T97, T98, T108, and T115 cell lines. The level of expression varied with changing distinctive tumorigenic properties [34].

Another noteworthy issue is the possibility of using PARP-1 as a potential target for therapy. PARP-1 inhibitors are mentioned as one of the treatment options for cancer—as they influence the stabilization of the genome and repair of DNA mutations [35]. PARP-1 inhibitors are already used in the treatment of certain types of cancers—ovarian, breast, and prostate [36,37]. In oral squamous cell carcinomas, overexpression of PARP-1 may serve as novel predictive biomarker for therapy responsiveness [38]. Research is also underway on the use of PARP-1 inhibitor in treatment other diseases, among others multiple sclerosis, rheumatoid arthritis, myocardial infarction, stroke, acute neuroinjury, and Parkinson’s disease [39,40]. Our demonstrated high PARP-1 expression in the examined tissues indicates a possible target for pharmacological therapy in UM. This study could be valuable for future introduction of new treatment regimens as it has already been shown on a UM patient-derived xenografts (PDXs) that PARP inhibitor olaparib significantly increased the efficacy of the alkylating agent dacarbazine in UM [41]. These results represent a very promising subject for further research into the treatment of UM.

## 5. Conclusions

Despite the great interest in the role of PARP in cancer formation, its role in UM is relatively poorly studied with only a few published reports. Our study shows that PARP-1 expression can be useful prognostic and predictive biomarker in UM. This study could also be valuable for future introducing new treatment regimens.

## Figures and Tables

**Table 1:** Clinicopathological characteristics of the study group.

Clinical Factors	No (%)
Gender	
Male	36 (39%)
Female	55 (61%)
Age	
≤ 63	43 (47%)
> 63	48 (53%)
iliary body involvement	
No	54 (59%)
Yes	37 (41%)
Iris involvement	
No	84 (92%)
Yes	7 (8%)
Irido-corneal angle involvement	
No	84 (92%)
Yes	7 (8%)
Intrascleral extension	
No	13 (14%)
Yes	78 (86%)
Extrascleral extension	
No	81 (89%)
Yes	10 (11%)
Tumor size	
≤15 mm	55 (60%)
>15 mm	36 (40%)
Mitoses *	
≤4	56 (63%)
>4	33 (37%)
Grade	
G1+G2	72 (79%)
G3	19 (21%)
Chromosome 3 loss **	
No	16 (24%)
Yes	51 (76%)
Metastases	
No	47 (52%)
Yes	44 (48%)

The total high PARP-1 expression (IRS ≥ 4) was statistically significantly associated with the loss of chromosome 3 (*p* = 0.008), higher histopathological grade (*p* = 0.022), and absence of intrascleral extension (*p* = 0.014). Higher expression was more common in male patients (*p* = 0.033). A trend of correlation between high total PARP-1 expression and bigger tumor size was also noted (*p* = 0.079). The overall list of results is presented in Table 2. * Mitotic index available for 89 patients. ** Chromosome 3 status available for 67 patients.

**Table 2:** Associations of PARP-1 total expression with clinicopathological parameters in uveal melanoma patients.

Clinical Factors	PARP-1 Low Expression (0–3)	PARP-1 High Expression (4–12)	*p*-value
Gender			
Male	9	27	0.033
Female	26	29	
Age			
≤ 63	20	23	0.135
> 63	15	33	
Ciliary body involvement			
No	24	30	0.156
Yes	11	26	
Iris involvement			
No	33	51	0.576
Yes	2	5	
Irido-corneal angle involvement			
No	1	6	0.171
Yes	34	50	
Intrascleral extension			
No	1	12	0.014
Yes	34	44	
Extrascleral extension			
No	32	49	0.560
Yes	3	7	
Tumor size			
≤15 mm	25	30	0.079
>15 mm	10	26	
Mitoses			
≤4	25	31	0.126
>4	10	23	
Grade			
G1 + G2	32	40	0.022
G3	3	16	
Chromosome 3 loss			
No	11	5	0.008
Yes	16	35	
Metastases			
No	19	28	0.691
Yes	16	28	

**Table 3:** Statistically significant correlations of intensity of reaction against PARP-1 with clinicopathological parameters.

Clinical Factors	PARP-1 Low Intensity (0–1)	PARP-1 High Intensity (2–3)	*p*-value
Gender			
Male	8	28	0.036
Female	24	31	
Intrascleral extension			
No	1	12	0.025
Yes	31	47	
Tumor size			
≤ 15 mm	23	32	0.039
> 15 mm	9	27	
Chromosome 3 loss			
No	10	6	0.017
Yes	15	36	

**Table 4:** Statistically significant correlations of percentage of positive cells with clinicopathological parameters.

Clinical Factors	PARP-1 low percentage (0–2)	PARP-1 high percentage (3–4)	*p*-value
Tumor size			
≤ 15 mm	33	22	0.036
> 15 mm	14	22	
Grade			
G1 + G2	40	32	0.024
G3	7	12	
Chromosome 3 loss			
No	11	5	0.033
Yes	24	27	

## Data Availability

The data presented in this study are available on request from the corresponding author.
